# Citation of non-English peer review publications – some Chinese examples

**DOI:** 10.1186/1742-7622-5-12

**Published:** 2008-09-30

**Authors:** Isaac CH Fung

**Affiliations:** 1 Department of Infectious Diseases Epidemiology, Imperial College London, UK

## Abstract

Articles published in English language journals with citations of non-English peer reviewed materials are not very common today. However, as epidemiologists are becoming more aware of data and information being readily available and accessible in the non-English literature, the question of whether non-English materials can be cited in English language journals and if so, how should they be cited, has become an increasingly important issue. Bringing together personal insights from the author's familiarity with both the English and Chinese language epidemiological literature and results from a survey on the use of citations of non-English peer reviewed materials across a sample of epidemiology and public health journals, this commentary discusses the different ways authors cite non-English articles in different English language journals and the different methods used by journals to handle non-Latin scripts (e.g. transliteration). This commentary will be useful to both epidemiologists and editors alike.

## Background

English has long been the *lingua franca *of the scientific world [1]. However, as demonstrated by the various articles in this thematic series of the *Emerging Themes in Epidemiology*, there are a substantial number of epidemiology and public health journals published in languages other than English today. However, for various reasons, these non-English journals struggle to survive in this competitive world. One possible reason for this is that articles in these journals are hardly cited, perhaps because of their perceived (or real) low quality and/or limited readership, which then contribute to a low impact factor (had they been fortunate enough to be indexed by Thomson Scientific), which then further discourages authors to submit high-quality research articles to these journals.

### The importance of citations

Citations are used in scholarly works to give credit to or acknowledge the influence of previous works, where credit is due. The annual *Journal Citation Reports *published by Thomson Scientific include a journal impact factor, which is "a ratio between citations and recent citable items published" [2]. It is calculated by dividing the number of cites received in a given year to articles published in the preceding two years by the number of articles published in those two years (citable items). For example, if a journal published 100 citable articles (as defined by Thomson Scientific) in 2006 and 2007, and it was cited 200 times in 2008 for those 100 articles, then the journal receives an impact factor of 2. The journal impact factor was never meant to be the sole basis for 'quality' ranking of journals or research outputs and its merits and weaknesses are open to continual discussion [3,4]. However, it has nevertheless become a significant part of research assessment exercises for universities and research institutions, simply because research outputs for individual researchers and institutions are often measured by the number of articles published in high-quality journals, which are in turn measured by their impact factors. Henceforth, it is understandable that journals whose articles attract more citations will be favoured by authors. Citations matter.

### Citation of articles published in languages other than English

There are a number of possible reasons why authors writing in English are less inclined to cite articles published in languages other than English. The authors may simply not be familiar with or proficient in those languages. In addition, these articles and journals may not be accessible to the authors. Given that references may be used to support ideas, suggest new ideas and verify accuracy, they may fear that readers of their English articles may not be able to read or access those articles and verify their ideas or accuracy. These reasons are legitimate concerns, for it is a reasonable assumption that a reader of an English article is more likely to have access to and be able to read an English article cited therein rather than a Chinese or Spanish one. These may also partly explain why some journal editors may prefer citations in English to one in other languages. Editors may find it difficult to find reviewers who can verify non-English citations. The fact that English has become the *lingua franca *of the international scientific community may seem to some as the *raison d'être *of a policy of not allowing citations from non-English materials.

In this thematic series, Liu *et al*. [5] draws our attention to the example of how artemisinin and its antimalarial properties, discovered by Chinese scientists and published in Chinese journals in the 1970s, were made known to the global scientific community at large after being reviewed in a top-tiered American journal in 1985. Since then, the English review article has received hundreds of cites. This observation on one hand acknowledged the status of English as the medium of communication of the international scientific community, while on the other hand suggests that important scientific discoveries have been published in non-English languages. The *a priori *assumption that scientific papers written in non-English languages are by default of a lower standard is unsound.

The effect of avoiding citations of non-English articles on the survival and development of non-English journals and thus academic career of our scientific colleagues working in the non-English-speaking world is seldom acknowledged and discussed, and warrants more in-depth studies in the future.

## Citations of non-English peer review articles in epidemiological and public health journals

To shed light on citations of non-English peer-reviewed articles that appear in articles submitted to English language epidemiology and public health journals, a simple survey was conducted on these journals. A total of 67 journals were contacted with a response rate of 38.8% (26/67). Of the 26 journals that responded, 23 agreed to participate. Of those 23 participating journals, 22 allowed authors to cite non-English materials in their papers. Twenty-one journals granted permission to have their responses cited as personal communications (see Table 1). The journal that did not permit citation of non-English materials and another journal of which information was preliminary, refused to be mentioned and they are not shown in Table 1.

**Table 1:** The responses from epidemiology and public health-related journals that participated in the survey and agree to have their responses quoted as personal communications^a^.

**Journals**	**Allow Citations of non-English material?**	**The format in which the citations must be written**	**Is a different format recommended for non-Latin characters e.g. Chinese?**
American Journal of Preventive Medicine	Y	A	"Must be romanized." (personal communication, Charlotte Seidman, 6 February 2008)
American Journal of Public Health	Y	A or B.	"AJPH does not allow non-Latin characters in references. For citations that references foreign sources, a translation [in brackets] must be provided after the original language." (personal communication, Brian Selzer, 20 December 2007)
Annals of Epidemiology	Y	A	No. Same format.
BMC Medicine	Y	A	"Not really applicable – we can include most characters. The limitations here are related to our ability to mark-up special characters into XML." (personal communication, Melissa Norton, 20 December 2007)*
BMC Public Health	Y	A	Same as *BMC Medicine*.
Central European Journal of Public Health	Y	A	No. Same format.
Clinical practice and epidemiology in mental health	Y	C	No. Same format.
Emerging Themes in Epidemiology	Y	A	ETE follows the style of *BMC Medicine*. However, regarding Chinese journal titles, ETE recommends that after spelling out the journal titles on pinyin (romanised Chinese), authors can include the English or Latin translations of the journal title for intelligibility for non-Chinese-speaking readers.
Epidemiology	Y	A, B or C "which ever of these the authors provide" (personal communication, Margaret McCann, 18, December 2007)**	"We do not allow the use of non-Latin characters." (personal communication, Margaret McCann, 18 December 2007)
Epidemiology and Infection	Y	A	"The journal title will be in Roman letters." (personal communication, Norman Noah, 20 February 2008)
European Journal of Cancer Prevention	Y	A	No. Same format.
European Journal of Cardiovascular Prevention and Rehabilitation	Y‡	A	"Not applicable." (personal communication, Phil Daly, 11 January 2008)
Injury Prevention	Y	C	"This issue has not arisen to date." (personal communication, Brian Johnston, 18 January 2008)
Journal of Public Health Management and Practice	Y	B	No. Same format.
Journal of Public Health Policy	Y	A, B or C (often C)	"Our authors have usually translated the reference from non-Latin characters so we have not tried to typeset non-Latin characters" (personal communication, Anthony Robbins, 27 December 2007)
Nature (and all journals with "Nature" in title, 38 journals in total, including Nature Clinical Practice journals) †	Y	D. "We follow the authors' usage. Authors generally supply the translation of the title rather than the original language, possibly because of PubMed listings or because foreign-language journals often provide an English-language abstract. If the author supplies a citation in the original language, we do not ask him/her to re-supply in English, but use the language supplied by the author." (personal communication, Maxine Clarke, 7 January 2008)	"Chinese titles are always supplied in English and our sub/copy editors add ' [in Chinese]' after the title if this is obviously the case (it isn't always obvious). We don't receive titles in, e.g., kanji or Cyrillic for our journals, but the Asia-Pacific edition of Nature has several additional translated pages at the front of each weekly issue (title, author names, summary of paper) for the local readership, and websites in Japanese, Korean and Chinese that contain additional material. These additional pages and websites are produced locally in the Asia-Pacific region, where the fonts are standard." (personal communication, Maxine Clarke, 7 January 2008)
NPG Academic Journals†	Y	D. "Like the Nature journals, we also tend to follow what is supplied by the author. If the author provides an English translation only then we would indicate this and specify the original language." "If the reference is supplied in the original language then we would publish as supplied (formatted to journal style)." (personal communication, Claire Andrews, 22 January 2008)	"We can also accommodate non-Latin characters, e.g. Chinese." (personal communication, Claire Andrews, 22 January 2008)
New England Journal of Medicine	Y	D." Titles of non-English can be given in the original language or in English. If the latter, a parenthetical note indicating the original language appears at the end of the citation" (personal communication, Sarah Fishkin, 20 December 2007)	No. "Citations to material published in non-Latin characters should be translated into English, with a parenthetical note indicating the original language at the end of the citation." (personal communication, Sarah Fishkin, 20 December 2007)
Preventive Medicine	Y	C	No. "We have never had this problem. We would keep the original characters if there is no official translation of the title and if no parts of the papers are available in English."
Proceedings of the National Academy of Science	Y	A	"We prefer that authors translate foreign titles into English, which helps to increase searchability of the references." (personal communication, Diane Sullenberger, 16 January 2008)
Tobacco Control §	Y	D "We would ask authors to translate the title of the paper/book/report into English but leave the name of the journal in the original language." (personal communication, Simon Chapman, 4 January 2008)	"We have never experienced this." (personal communication, Simon Chapman, 4 January 2008)
Transactions of the Royal Society of Tropical Medicine and Hygiene	Y	A	No: "not for Greek, Russian, Bulgarian, Korean, Thai, Chinese, Japanese, Arabic etc. This does not imply any judgement as to the standard of publications in these or any other languages." (personal communication, Bohumil S. Drasar, 15 January 2008)

^a ^Two journals replied and agreed to participate in this survey, but refused to have their replies cited as personal communication. Therefore, they are not presented in this table.

* The answer for BMC Medicine applies to all BMC-series journals , but does not necessarily apply to all other non-BMC series journals that BioMed Central publishes.

** According to Margaret McCann of *Epidemiology *(personal communication, 18 December 2007), all journals published by Lippincott Williams & Wilkins adhere to the 10^th ^edition of *AMA Manual of Style: A Guide for Authors and Editors*.

‡ European Journal of Cardiovascular Prevention and Rehabilitation's answer was 'a qualified "yes". As an English language publication the editors need to consider the accessibility and value of these sources so that readers also can also make their own assessment of the study results presented' (personal communication, Phil J. Daly, 10 January 2008).

† For the response from *Nature *and NPG academic journals, we only counted as one journal (*Nature*) in the denominator of 67 journals. A few other journals published by the NPG were surveyed too were counted individually in the denominator. For a list of NPG journals, please visit .

§BMJ Journals have similar policies across all their journals (personal communication, Claire Folkes, 16 January 2008).

### Methods

Epidemiology and public health journals were selected based on the journal title and scope. Further journals were identified by searching the PubMed Journals database for all English language journals with "epidemiology" or "public health" in the title. Journals no longer in regular publication and journals without a website were excluded. Five top-tier scientific or medical journals that are of relevant to the practice of epidemiology and public health but not fitting the above criteria were included. A preliminary survey of a sample of epidemiology and public health journals was carried out to determine key themes related to citation of non-English materials and this information was used to inform the final questionnaire design (see Endnote). Then each of the selected journals' editorial boards was contacted via email, and was invited to participate in this short survey. Responses from editorial boards that agreed to participate in the survey but did not grant permission to quote their responses as personal communications were only used in the aggregated measures.

### Results

Among the 23 participating journals, 22 allowed authors to cite non-English materials. Eleven of these 22 journals ask authors to translate the citation into English followed by the original language in parentheses or brackets (option A); one recommends that the non-English language title be given first, followed by the English translation in brackets (option B); and three print the original title only (option C). A number of other journals either allow more than one of the above options or follow what is supplied by the author. Regarding the question as to whether a different format is recommended for non-Latin characters, e.g. Chinese, while some journals follow exactly the same format as languages of the Latin alphabet, some require romanisation of non-Latin characters. For details, please refer to Table 1.

The reason for one journal not allowing citation of non-English materials was that 'English is considered to be the international language of science' (the journal editor did not grant me permission to quote his/her response as a personal communication and thus I was not allowed to disclose the identity of the journal). One journal editor expressed personal reservation on allowing citations of non-English materials as non-English references cannot be verified or accessed by the reader and it is difficult to find reviewers for non-English citations (i.e. options B and C in question 1), although his journal *does *allow citation of non-English materials as a policy (personal communication, Brian D. Johnston, *Injury Prevention*, 18 January 2008).

### Discussion

Twenty-two journals among the 23 journals participating in the survey allowed authors to cite non-English materials in their papers. The majority of these journals preferred the title to be translated into English, followed by indicating the original language in brackets or parentheses, e.g. [in Chinese]. Some journals follow the same format for references of any language while some require references in languages of the non-Latin alphabet or characters to be romanised. Understandably, for those journals that only print the translated title in English, this issue matters little except for the journal title. However, for those journals that print the original article title only, or those that print both the original and its translation, this might be a matter of concern. These issues will be discussed in sections below.

The intention of this survey is more qualitative than quantitative and the figures given above are not intended to be used as quantitative estimates for analytical purposes. This survey provides us with examples on how English language journals handle non-English citations despite a low response rate which may render the survey results non-representative. A potential bias would be that those journals participating in this survey were more likely to agree with the intention of the author and the purposes of this survey while those that were sceptical declined to take part. Consideration of potential adverse publicity might also discourage participation. The preliminary survey that was conducted might have engendered distrust among some journal editors as I had not explicitly expressed my intention to conduct a survey when we first approached some of the journals by emails. I apologised to the editors concerned and when I conducted my final survey, I stated that I would like to invite the editors of the journals to participate in a survey and explicitly asked for permission to cite their responses as personal communications. All the responses that are presented here were granted permission by the journal editors concerned.

## Non-English journal articles: how should we cite?

To help better elucidate the practice of citation of non-English articles in English language journals, in the following sections I will discuss the handling of non-English article titles, non-Latin scripts, transliteration and non-English journal titles in non-Latin scripts. I will conclude with some additional notes and comments.

### How do we handle non-English article titles?

The American Medical Association (AMA)'s *AMA Manual of Style: A Guide for Authors and Editors *(10^th ^edition) [6] suggests several options for non-English-language article titles:

(1) as they originally appeared, without translation:

"1. Richartz E, Schott KJ, Wormstall H. Psychopharmakotherapie bei Demenzerkrankungen. *Drsch Med Wochenschr*. 2004;129(25/26):1434–1440.

2. Ohayon MM. Prevalencia y factores de riesgo de cefaleas matinales en la población general. *Arch Intern Med Ed Espanol*. 2004;1(1):41–47. Originally published, in English, in: *Arch Intern Med*. 2004;164(1):97–102." [6]

(2) translated into English, with the original language indicated in brackets and following the title:

"3. Miyazaki K, Murakami A, Imamura S, et al. A case of fundus albipunctatus with a retinol dehydrogenase 5 gene mutation in a child [in Japanese], *Nippon Ganka Gakkat Zasshi*. 2001; 105(8):530–534." [6]

(3) provided in its original language, followed by the English translation in brackets:

"4. Camici M. Cancro prostático e screening con test PSA [Prostate cancer and prostate-specific antigen screening]. *Minerva Med*. 2004;95(1):25–34." [6]

The US National Library of Medicine (NLM), in *Citing Medicine: the NLM style guide for authors, editors, and publishers *(2^nd ^edition) [7], states that the title is translated to English and enclosed in square brackets. This is similar to option two in the *AMA Manual of Style*, except that the original language of the article is shown after the location (pagination), followed by a full stop. One of the examples given in [8] is:

"Itabashi M, Yoshida K, Kameoka S. [Sentinel node navigation surgery for colorectal cancer]. Gan To Kagaku Ryoho. 2005 Apr;32(4):557–60. Japanese."

However, the NLM also suggests that the original language title or romanized title should be placed before the translation when possible, i.e. option three in the *AMA Manual of Style*. NLM recommends that unless the conventions of a particular language requires otherwise, only the first word of the title, proper nouns, proper adjectives, acronyms and initialisms is capitalised. Also, they recommend that in the titles, diacritics, accents and other special characters should be ignored to simplify rules for publication in English. An example is given in [8]:

"Neue Nifedipin-Zubeitung ermoglicht tagliche Einmalgabe [New nifedipine preparation makes single daily dose possible]. Fortschr Med. 1997 Nov 30;115(33): [following p. 54]. German."

Among the epidemiology and public health-related journals that replied to the questionnaire, the majority used option two above (Table 1).

### How do we handle non-Latin scripts?

Regarding references in languages that use a non-Latin script, we can consider the following questions:

1) Are official English translations available or not?

2) Should we use translation or transliteration or both?

3) How do we handle journal titles?

Nowadays many journals published in non-English languages offer table of contents and abstracts in English. However, what about those journal articles where there is no translation to English, including the title of the articles? An article title in a non-English language, e.g. Chinese, can probably be translated to English in more than one way. Is there a way of helping to prevent confusion here?

### Transliteration

One solution to this problem is transliteration which involves using Latin alphabets (known as romanisation or Latinisation) [9,10], followed or preceded by the English translation. This allows for accurate interpretation to readers who are proficient in the original language, and allows the average English reader (who is not proficient in the original language) a sufficient level of understanding. However, even transliteration can be problematic, for example, mainland China and Taiwan follow different schemes of romanisation of Chinese into English [11]. Currently the NLM adopts the *pinyin *scheme of transliteration, formally called *Hanyu Pinyin*, which is the official standard in mainland China and is also adopted by the International Organisation for Standardisation as well as the United States Library of Congress [12,13]. Recently the NLM has finished its multi-year project to convert its bibliographic records into *pinyin *from the old Wade-Giles romanisation scheme, which was popular in the West before the United States established diplomatic ties with the People's Republic [14], while some early records in Pubmed and Medline might still have been left unconverted [15]. In Taiwan, the Wade-Giles scheme was the *de facto *standard [14], while in 2002, the Taiwanese government approved a new official standard known as *Tongyong pinyin*, which is a revised version of the mainland Chinese *pinyin *system [16]. Chemical Abstracts has adopted the *pinyin *scheme for transliteration of Chinese authors' names in mainland Chinese publications since 1982 but retained the Wade-Giles scheme for Taiwanese publications [17]. It would be beneficial if journals can adopt a clear policy on matters of transliteration should there exist more than one scheme of transliteration currently in use for a given language into English.

### Handling citation of journal titles in non-Latin scripts

Translation of journal titles requires further attention. Even if the article titles are translated differently in two different citations, as long as the readers can get hold of the right journal and its volume and page numbers, they can tell that the two citations refer to the same journal article. However, if confusion of journal titles arises, this will be problematic. PubMed Journals Database [18] uses transliteration of journal titles written in non-Latin scripts. Take for example, the *Chinese Journal of Epidemiology *is transliterated as *Zhonghua Liu Xing Bing Xue Za Zhi*. This avoids confusion between journals in different languages but with similar names. Thus if a journal is indexed in PubMed, the PubMed/Medline format should be followed. To help make this transliterated journal title more intelligible and informative, it may be even better to have its English title (if it had one) available in parentheses or brackets following the transliteration, like *Zhonghua Liu Xing Bing Xue Za Zhi (Chinese Journal of Epidemiology)*. However, extra care needs to be taken for journals that are not indexed in PubMed. Take again for example, Chinese journals. There is a Medline-indexed medical journal, *Journal of Huazhong University of Science and Technology (Medical Sciences) *(NLMID: 101169627), that publishes articles in either English or German. However, it has a sister journal published in Chinese that is not indexed in PubMed and the Chinese title which if translated, will be literally the same as that of the English journal. Therefore, the journal in Chinese has a Latin title to avoid confusion: *Acta Medicinae Universitatis Science of Technologiae Huazhong *(cf. footnote 6 in Table 1 in [19]). If for some reason, the journal title of the latter is translated into English without any qualifications, this will lead to much confusion and possibly citation errors. Thus, for journal titles in non-Latin-script languages, it may be a good idea to either print it in the original language or to transliterate it into the Latin alphabet, and in either way followed by an English or Latin translation (it would be best if the translation is provided by the journal publisher).

### Additional notes on citations in languages with non-Latin scripts

With the advancement of computer technology, the problem traditionally associated with typesetting might be solved in the near future. A journal editor who responded to the survey commented that the BMC journals could include most characters. Their limitations are related to their ability to mark-up special characters into XML (personal communication, Melissa Norton, *BMC Medicine*, 20 December 2007). The Asian-Pacific edition of *Nature *contains several additional translated pages at the front of each weekly issue (title, author names and summary of paper) for the local readership, and websites in Japanese, Korean and Chinese that contain additional material. As these additional pages and websites are produced locally in the Asia-Pacific region, they follow the font standard used locally. *Nature *is currently investigating the possibility of adding Chinese characters (known as *kanji *in Japanese) to their fonts in their US/UK editions, with a view to offer Chinese authors the option of publishing their names in Chinese characters in addition to their English transliteration in their research papers (personal communication, Maxine Clarke, *Nature*, 7 January 2008). This laudable attempt is worth the attention by other journals and publishers. By providing the original names in Chinese characters alongside their English transliteration, this in the long run would help avoid confusion of authorship in citations, a common phenomenon among East Asian authors with popular surnames. For example, by typing the popular Chinese name "Chen C [au]" and popular Korean name "Kim S [au]" in PubMed, 14652 and 19716 items are found respectively (as of 7 February 2008)! With the growing use of the Unicode standard in the internationalization and localization of computer software, and its implementation in recent technologies including XML and the Java programming language [20], creating an interface that can properly display various writing systems of the world [21] is now possible.

## Some final comments

A journal editor noted that his journal "has not formulated rules on these matters" and what it tries to do is "what is useful to our readers, who may want to find, read, and even translate referenced articles" (personal communication, Anthony Robbins, *Journal of Public Health Policy*, 27 December 2007). The intention of writing this commentary is to raise awareness among editors and authors alike of various problems that may arise when authors cite non-English peer review materials in their articles, and review the different solutions available to solve these problems. I am not advocating any standardised format of citation of non-English peer review materials. I would suggest, however, transparency in editorial policy regarding this matter. I searched the journal websites before approaching the editors (in the survey) by email. However, on many of these websites, no information regarding citations of non-English materials was found. Had I been preparing a paper with citations of many non-English articles, what would I do?

I would like to raise some questions for our fellow editors working for other journals to ponder – would some journals reply differently to a potential author's enquiry rather than to a survey? Had I intended to submit an article with citations of Chinese articles, would my article be rejected by some journals simply because I cited Chinese articles in my paper? Or would I be asked to remove those citations simply because some journal editors were unable to find reviewers who could verify them? Would I experience 'discrimination' simply because English is now the international language for science and citing research articles published in Chinese journals would deem to be unnecessary?

I write this commentary with a personal belief that it would be beneficial to our field if we epidemiologists could also make use of data published in non-English languages. Credit should be given to our fellow colleagues who work and publish in the non-English-speaking world through citing their original research articles published in non-English journals. As a managing editor of an epidemiology journal who has come across submitted articles with citations in non-English languages, and as an author who has previously written review papers that cited Chinese articles [22,23], I sincerely hope that this commentary will be beneficial to fellow editors working for other journals and their potential authors who may cite non-English materials in their papers.

## Abstracts in non-English languages

The abstract of this commentary has been translated into the following languages by the following translators (names in brackets):

• Chinese – simplified characters (The author) [see Additional file 1]

• Chinese – traditional characters (The author) [see Additional file 2]

• French (Mr. Philip Harding-Esch) [see Additional file 3]

• Spanish (Ms. Annick Bórquez) [see Additional file 4]

## Endnote. Survey on the citation on non-English material

1. Does your journal allow the citation of non-English material?

If NO – what are the reasons for this policy? e.g. (more than one may apply)

A. "Non-English literature is considered to be of inferior quality"

B. "Non-English references cannot be verified or accessed by the reader"

C. "Difficulties in finding reviewers for non-English citations"

D. Other (it would be helpful if you could provide a reason).

Please jump to question 4.

If YES, please answer questions 2, 3 and 4

2. What format must the citation be written?*

A. "Translate into English followed by original language in parentheses."

B. "Provide both the original and translated title side by side."

C. "Provide the citation in the original language only"

D. Other (please provide details)

3. Is a different format recommended for non-Latin characters e.g. Chinese?

Please provide details:

4. Do you grant your permission to quote your response as personal communication in our article?

*Answers A, B and C of question 2 refer to the options available in the *AMA Manual of Style *recommendations. The wording used in the original questionnaire might not be as clear as it should be. A better wording for Answer A would be "Translate into English followed by *indicating *the original language in parentheses". Answer B could be re-written as "The non-English language title be given first, followed by the English translation, in brackets" as given in the *AMA Manual of Style *or "vice versa".

## Competing interests

ICHF is currently one of the managing editors of *Emerging Themes in Epidemiology*.

## Authors' contributions

ICHF conceived the idea of this project, collected the data, did the analysis and wrote this commentary.

## Funding

ICHF received no funding for this project.

## Disclaimer

The opinion expressed in this article only represents that of the author and does not necessarily represent that of the editorial board of ETE.

## Supplementary Material

Additional file 1 Abstracts in Chinese (simplified characters). Abstracts in Chinese (simplified characters) Click here for file

Additional file 2 Abstracts in Chinese (traditional characters). Abstracts in Chinese (traditional characters). Click here for file

Additional file 3 Abstracts in French. Abstracts in French. Click here for file

Additional file 4 Abstracts in Spanish. Abstracts in Spanish Click here for file
