# Supplementary material for: Citation of non-English peer review publications – some Chinese examples
Source: Emerg Themes Epidemiol. 2008 Sep 30;5:12. doi: 10.1186/1742-7622-5-12 (PMC2570362; doi:10.1186/1742-7622-5-12)
Supplement: Additional file 1 — Abstracts in Chinese (simplified characters). Abstracts in Chinese (simplified characters) [file 1742-7622-5-12-S1.pdf]

Simplified Chinese / 简体中文

评论

经过同行评审的非英语刊物文献的引用——一些中文例子

作者：冯俊熙　(Isaac Chun-Hai FUNG)

摘要

如今并不常见，在英语期刊上发表的文章里，引用经过同行评审的非英语文献。然而，当流行病学家日渐注意到此类文献中的资料与信息可供随时获取时，可否在英语期刊中引用非英语文献，以及如果可以，该如何引用的问题，成为了一个愈来愈重要的议题。这篇评论系具熟悉中英文流行病学文献的作者的个人洞见，并结合了一项就流行病学及公共卫生期刊有关引用经过同行评审的非英语文献的调查结果；本文讨论作者在不同的英语期刊上引用非英语文章的不同方法，以及期刊处理非拉丁字母文字的不同方法（如：音译）。相信这篇评论会对流行病学家和编辑都有所启益。

（由作者本人翻译）
