# Supplementary material for: Citation of non-English peer review publications – some Chinese examples
Source: Emerg Themes Epidemiol. 2008 Sep 30;5:12. doi: 10.1186/1742-7622-5-12 (PMC2570362; doi:10.1186/1742-7622-5-12)
Supplement: Additional file 2 — Abstracts in Chinese (traditional characters). Abstracts in Chinese (traditional characters). [file 1742-7622-5-12-S2.pdf]

Traditional Chinese / 繁體中文

評論

經過同行評審的非英語刊物文獻的引用──一些中文例子

作者：馮儁熙　(Isaac Chun-Hai FUNG)

摘要

如今並不常見，在英語期刊上發表的文章裡，引用經過同行評審的非英語文獻。然而，當流行病學家日漸注意到此類文獻中的資料與信息可供隨時獲取時，可否在英語期刊中引用非英語文獻，以及如果可以，該如何引用的問題，成爲了一個愈來愈重要的議題。這篇評論系具熟悉中英文流行病學文獻的作者的個人洞見，並結合了一項就流行病學及公共衛生期刊有關引用經過同行評審的非英語文獻的調查結果；本文討論作者在不同的英語期刊上引用非英語文章的不同方法，以及期刊處理非拉丁字母文字的不同方法（如：音譯）。相信這篇評論會對流行病學家和編輯都有所啓益。

（由作者本人翻譯）
