# Supplementary material for: Citation of non-English peer review publications – some Chinese examples
Source: Emerg Themes Epidemiol. 2008 Sep 30;5:12. doi: 10.1186/1742-7622-5-12 (PMC2570362; doi:10.1186/1742-7622-5-12)
Supplement: Additional file 3 — Abstracts in French. Abstracts in French. [file 1742-7622-5-12-S3.pdf]

French / Français

Commentaire

La citation de revues à comité de lecture publiées dans des langues autres que l'anglais : quelques examples chinois

Auteur : Isaac Chun-Hai Fung

Résumé

Peu nombreux sont les articles publiés actuellement dans les revues en anglais citant des publications à comité de lecture écrites dans des langues autres que l'anglais. Cependant, au fur et à mesure que les épidémiologistes se rendent compte des données et des informations qui sont facilement disponibles et accessibles dans la littérature publiée dans d'autres langues, il est de plus en plus important de régler la question suivante : peut-on citer la documentation publiée dans des langues autres que l'anglais et si oui, comment faire ?

Ce commentaire réunit les perspectives personnelles de l'auteur, qui connaît la littérature épidémiologique en langue chinoise comme celle en langue anglaise, aux résultats d'une étude sur la manière dont les documents à comité de lecture publiés dans des langues autres que l'anglais sont cités dans un échantillon de revues d'épidémiologie et de santé publique. On y examine les différentes manières dont les

auteurs citent les articles publiés dans des langues autres que l'anglais dans diverses revues de langue anglaise, et les diverses méthodes employées par les revues pour traiter les documents en caractères non-latins (par exemple, la translittération). Ce commentaire sera utile aux épidémiologistes tout comme aux rédacteurs.

Traduit de l'anglais par Philip Harding-Esch
