# Supplementary material for: Citation of non-English peer review publications – some Chinese examples
Source: Emerg Themes Epidemiol. 2008 Sep 30;5:12. doi: 10.1186/1742-7622-5-12 (PMC2570362; doi:10.1186/1742-7622-5-12)
Supplement: Additional file 4 — Abstracts in Spanish. Abstracts in Spanish [file 1742-7622-5-12-S4.pdf]

Spanish / Español

Comentario

Citaciones de publicaciones revisadas por pares escritas en otros idiomas que el inglés - algunos ejemplos chinos.

Autor: Isaac Chun-Hai Fung

Resumen

Hoy en día son pocos los artículos publicados en revistas en inglés que citan material escrito en otros idiomas. Sin embargo, conforme los epidemiólogos se van dando cuenta de la disponibilidad y accesibilidad de información y de datos en la literatura científica en otros idiomas, se presenta la pregunta siguiente: ¿es posible citar estos materiales en revistas en inglés? Y en caso de serlo, el cómo hacerlo se ha convertido en un problema cada vez más importante. Reuniendo percepciones del autor, que resultan de su familiaridad con la literatura en epidemiología en chino y en inglés, con resultados de una encuesta acerca del uso de citaciones de materiales escritos en idiomas otros que el inglés por una muestra de revistas de epidemiología y salud pública, este comentario analiza las diferentes formas en las que los autores citan artículos en otros idiomas en diferentes revistas en inglés y los distintos métodos empleados por las revistas para manejar caracteres que no son

latinos (Ej. Transliteración). Este comentario le será útil tanto a epidemiólogos como a editores.

Traducido al español por Annick Bórquez
